# Mediating Role of Health Literacy in Relationship Between Frailty and Medical Costs in Community-Dwelling Older Adults

**DOI:** 10.3390/healthcare13111331

**Published:** 2025-06-03

**Authors:** Hee-Sun Kim, Jinhee Kim

**Affiliations:** 1 National Evidence-Based Collaborating Agency, Seoul 04933, Republic of Korea; hskim7336@neca.re.kr; 2 Department of Nursing, Chosun University College of Medicine, Gwangju 61452, Republic of Korea

**Keywords:** older adults, frailty, health literacy, medical cost, mediating role

## Abstract

This study aims to examine the mediating effects of health literacy on the relationship between frailty and medical costs among community-dwelling older adults. **Methods:** This study conducted a secondary data analysis of the research data that were constructed by linking the Korean Frailty and Aging Cohort Data (KFACD) and the National Health Insurance Database (NHID). Frailty was measured using the Modified Fried Phenotype. Medical costs were calculated using insurance-covered medical costs, including both inpatient and outpatient medical costs, from January 1 to December 31 of the year when the participants were enrolled in the Korean Frailty and Aging Cohort Study. Health literacy was assessed using questions from the Behavioral Risk Factor Surveillance System (BRFSS) conducted by the US Centers for Disease Control and Prevention. To examine the mediating role of health literacy in the relationship between frailty and medical costs, Baron and Kenny’s method was used. Linear regression was applied to estimate the association between frailty and health literacy, and Poisson regression was used to model the relationship between frailty, health literacy, and medical costs. **Results:** Frailty showed a negative correlation with health literacy (r = −0.27, *p* < 0.001) and a positive correlation with medical costs (r = 0.15, *p* < 0.001). Health literacy had a negative correlation with medical costs (r = −0.07, *p* = 0.008). We verified that health literacy played a partial mediating role in the relationship between frailty and medical costs. **Conclusions:** To reduce medical costs in older adults, intervention measures to improve health literacy as well as prevention and management measures for frailty should be considered simultaneously. In addition, primary medical institutions’ active participation in such projects is needed.

## 1. Introduction

South Korea is a country with one of the fastest aging populations worldwide. The proportion of the older adult population aged 65 or older in South Korea is expected to increase from 12.8% in 2015 (6.38 million people) to 25.3% in 2030 (12.98 million people), representing more than a 100% increase over 15 years [1]. In general, older adults have health problems such as chronic diseases, physical and cognitive decline, disabilities, and frailty and, thus, require more utilization of medical resources, which becomes a burden on the national economy [2]. In addition, as the health insurance coverage under the national health insurance system in the Republic of Korea is low, excessive utilization of medical services can be a burden on individuals and can cause poverty among older adults [3]. South Korea has a universal single-payer health insurance system, but partial coverage for some services may lead older adults to forgo care due to out-of-pocket costs. Therefore, national and individual efforts are required to reduce medical costs for older adults.

The rapidly aging population has increased interest in healthy life expectancy and healthy aging, and the concept of frailty as a factor that threatens older adults’ health has been gaining attention [4]. Frailty is a multifactorial clinical syndrome of a nonspecific condition in which older adults’ physical vulnerability to stressors increases and their physiological reserves decline, thereby reducing resistance to stress. Frailty is associated with increased use of healthcare resources and costs, as well as adverse health outcomes such as falls, disability, institutionalization, and death [5,6,7,8,9,10]. These patterns have also been observed among older adults in South Korea [4]. Healthcare costs in community-dwelling robust older adults were reduced compared to frail older adults and pre-frail older adults, and an increase in the frailty index was proportional to an increase in medical costs [9]. Therefore, to prevent negative health outcomes due to frailty and reduce medical costs, efforts to prevent and manage frailty are critical.

The increasing prevalence of frailty due to the rapidly growing older adult population leads to a focus on health literacy as one of the preventive approaches to functional decline in older adults [4,11]. Health literacy is defined as the cognitive and social skills to read, understand, evaluate, and use accurate health information to make informed health-related decisions and promote good health [12]. As health literacy levels decline with age, health literacy barriers are an area that requires special attention for older adults who frequently use healthcare and health-related information [13]. Previous studies have reported that low health literacy was a barrier factor to exercising control over health management and was associated with poor health outcomes, including death [14,15]. In addition, low health literacy was related to frailty progression in community-dwelling older adults and to an increase in healthcare utilization and medical costs among them [16,17]. To support the efficient use of medical resources and healthy aging in older adults, it is necessary to pay attention to improving health literacy levels.

Many previous studies [9,10,17] have reported the relationship between frailty and medical costs. However, studies analyzing the mediating effects of health literacy on the relationship between frailty and medical costs are scarce. Although a previous study [11] conducted in South Korea reported that health literacy had a moderating effect on the relationship between frailty and catastrophic health expenditures, the results were limited to a pre-frail group and, thus, required confirmation. Catastrophic health expenditures refer to out-of-pocket medical costs that exceed a household’s ability to pay and cause financial hardship. Against this backdrop, this study aims to examine whether health literacy plays a role in the relationship between frailty and medical costs among community-dwelling older adults, considering health literacy as a potentially modifiable factor. Although health literacy may influence health outcomes in earlier stages of aging, frailty progression often leads to reduced cognitive and functional capacity that can further compromise health literacy. This bidirectional relationship has been acknowledged, and our study considers one possible pathway as a hypothesis to be tested.

## 2. Materials and Methods

### 2.1. Study Design

This study is a cross-sectional secondary data analysis of research data provided by the National Evidence-Based Healthcare Collaborating Agency (NECA), which were constructed by linking Korean Frailty and Aging Cohort Data (KFACD) [18] and the National Health Insurance Database (NHID). Given the cross-sectional nature of the design, this study aims to examine associations rather than infer causal relationships among the variables. In this study, frailty was considered the independent variable, medical costs as the dependent variable, and health literacy as the mediating variable.

### 2.2. Data Source

This study used data from the KFACD [18] linked with the NHID (2016–2017). The KFACD contains data created from the Korean Frailty and Aging Cohort Study (KFACS) in 2016–2017, in which 3011 community-dwelling older adults aged 70–84 were enrolled, and the main survey content included frailty, health status, health behavior, health literacy, and health-related quality of life. The NHID for the participants enrolled in the KFACS in 2016 included both inpatient and outpatient medical costs from 1 January to 31 December 2016, while the NHID for those enrolled in 2017 included both inpatient and outpatient medical costs from 1 January to 31 December 2017. In this study, data from 1538 individuals without missing values for the main variables of interest were utilized from the KFACD. This study utilized anonymized data from 1538 individuals without missing values for the main variables of interest.

Data analysis was conducted from 29 August to 6 September 2024.

### 2.3. Measurements

#### 2.3.1. General Characteristics Variables

The general characteristics variables included in this study are gender, age, spouse, educational level, economic activity, household monthly income, self-rated health status, smoking, and drinking available in the KFACD [18].

#### 2.3.2. Frailty

In this study, frailty was measured using the modified version of Fried’s frailty phenotype, which was modified from the data from the Cardiovascular Health Study and has been validated in the Asia-Pacific region, including South Korea [19,20]. Fried’s frailty phenotype comprises five components: weight loss, weakness, exhaustion, slowness, and low physical activity. Each fulfilled component is assigned a value of 1, and each unfulfilled component is assigned a value of 0 [19]. If the score as calculated by the sum of values for each component is 0, the participant is defined as a robust older adult with a value of 1. If the score or the sum of values for each component is 1–2, the participant is defined as pre-frail with a value of 2, and if the score or the sum of values for each component is 3–5, the participant is defined as frail with a value of 3 [19,21]. Based on the total score, participants were categorized into three levels of frailty status (robust, pre-frail, and frail), and this ordinal variable was analyzed as a continuous variable in the regression models.

#### 2.3.3. Medical Costs

Medical costs are national insurance-covered medical costs, including both inpatient and outpatient medical costs (patient out-of-pocket payment + insurer payment), from 1 January to 31 December of the year when the participants were enrolled in the KFACS.

#### 2.3.4. Health Literacy

Health literacy was measured using three items in the Behavioral Risk Factor Surveillance System developed by the Center for Disease Control and Prevention in the US [21]. Each item is rated on a 4-point Likert scale, ranging from 1 point for “very difficult” to 4 points for “very easy”, and the total score was calculated by summing up scores for each item. The total score ranges from 3–12 points, and a higher score indicates higher health literacy [21]. In previous research [22], the reliability of this tool was reported as Cronbach’s alpha = 0.87, while in the present study, it was Cronbach’s alpha = 0.88.

### 2.4. Data Analysis

The general characteristics of the participants were presented as frequencies and percentages, and the levels of frailty, health literacy, and medical costs were presented as means and standard deviations. A t-test or one-way analysis of variance was performed to determine differences in levels of frailty, health literacy, and medical costs according to the general characteristics of the participants. Pearson’s correlation coefficient was utilized to examine the relationships among frailty, health literacy, and medical cost. To identify the mediating role of health literacy in the relationship between frailty and medical costs, we used Baron and Kenny’s three-step mediating effect verification method [23,24]. In the regression analysis of each step, the general characteristic variables for which statistically significant differences in the dependent variables were identified were adjusted. Medical costs, originally measured in Korean Won (KRW), were highly skewed and therefore log-transformed for analysis using a Poisson regression model. Accordingly, the regression coefficients reflect changes in the log of medical costs and should be interpreted as proportional changes rather than absolute differences in monetary value. Data were analyzed using the SAS software version 9.4 (SAS Institute Inc., Cary, NC, USA). 

## 3. Results

### 3.1. Characteristics of Participants

Of all participants, 61.1 percent were male. The age distribution of the study participants showed that the largest group was aged 70–75 years (50.7%), followed by those aged 76–80 years (34.1%) and 81–84 years (15.3%). Moreover, 27.0% of the respondents did not have a spouse. The proportion of individuals with an educational level of elementary school or lower was the highest at 39.4%, while those with a middle school education were the lowest at 14.9%. The proportion of those who were unemployed in terms of economic activity was 75.0%. The most common monthly household income was KRW 1 million at 39.0%, followed by KRW 1–2 million at 24.5% and KRW 3 million at 20.6%. The proportion of those who self-rated their health status as healthy was 73.0%. Among the study participants, 49.6% were non-smokers, and 43.4% were ex-smokers. The most common drinking pattern was non-drinking, accounting for 30.7%, followed by drinking less than once a month (29.3%) and drinking 2–4 times a month (15.7%) (Table 1).

### 3.2. Frailty Based on Participants’ Characteristics

The frailty level of the study participants was 1.89 ± 0.55, and statistically significant differences were present in gender (t = 7.01, *p* = 0.008), age (F = 37.82, *p* < 0.001), educational level (F = 5.27, *p* = 0.001), household monthly income (F = 8.67, *p* < 0.001), self-rated health status (t = 79.32, *p* < 0.001), and drinking (F = 4.09, *p* = 0.003). The frailty level was higher in men (1.91 ± 0.51). Among age groups, it was highest in those aged 81–84 years (2.13 ± 0.57), followed by those aged 76–80 years (1.91 ± 0.54). In terms of educational level, those with an elementary school education or less had the highest frailty level (1.95 ± 0.56), followed by those with a middle school education (1.85 ± 0.56). For household monthly income, those earning less than KRW 1 million had the highest frailty level (1.97 ± 0.57), followed by those earning KRW 1–2 million (1.87 ± 0.51). For self-rated health status, those who rated themselves as unhealthy had a higher frailty level (2.08 ± 0.56), and in terms of drinking, non-drinkers had the highest frailty level (1.95 ± 0.56) (Table 1).

### 3.3. Health Literacy Based on Participants’ Characteristics

The health literacy level of the study participants was 8.59 ± 2.62, and statistically significant differences were present in gender (t = 143.16, *p* < 0.001), age (F = 33.68, *p* < 0.001), marital status (t = 101.62, *p* < 0.001), educational level (F = 137.25, *p* < 0.001), household monthly income (F = 67.94, *p* < 0.001), self-rated health status (t = 147.66, *p* < 0.001), smoking (F = 28.31, *p* < 0.001), and drinking (F = 7.22, *p* < 0.001). The health literacy level was higher in males (9.20 ± 2.41). In terms of age, the highest health literacy level was observed in the 70–75 years age group (9.02 ± 2.46), followed by the 76–80 years age group (8.38 ± 2.58). The health literacy level was higher among those with a spouse (8.98 ± 2.50), and the highest level was observed among individuals with a college degree or higher (10.07 ± 2.05). In terms of household monthly income, the highest health literacy level was observed in those with an income of KRW 3 million or more (9.81 ± 2.13), while the lowest was found in those with an income of less than KRW 1 million (7.57 ± 2.73). In terms of self-rated health status, those who rated their health as healthy had the highest health literacy level (9.06 ± 2.47). Among smoking categories, ex-smokers had the highest health literacy level (9.10 ± 2.40). For drinking frequency, those who drank 2–4 times per month had the highest health literacy level (9.17 ± 2.55) (Table 1).

### 3.4. Medical Cost Based on Participants’ Characteristics

The medical cost for the study participants was 1,605,633 ± 2,761,270 Korean won (KRW), and statistically significant differences were present in self-rated health status (t = −16.97, *p* < 0.001) and drinking (F = 6.21, *p* < 0.001). In terms of self-rated health status, the medical cost was highest for those rated as unhealthy (KRW 2,079,588 ± 3,112,941), and in terms of drinking, the highest medical cost was observed among non-drinkers (KRW 2,065,895 ± 3,481,819) (Table 1).

### 3.5. Correlations Among Frailty, Health Literacy, and Medical Cost

Frailty and health literacy had a statistically significant negative correlation (r = −0.27, *p* < 0.001). Frailty and medical cost had a statistically significant positive correlation (r = 0.15, *p* < 0.001). Health literacy and medical cost had a statistically significant negative correlation (r = −0.07, *p* = 0.008) (Table 2).

### 3.6. Mediating Effect of Health Literacy on the Relationship Between Frailty and Medical Cost

To examine the mediating effect of health literacy on the relationship between frailty and medical cost, a three-step regression analysis was conducted [23] (Table 3). To examine the presence of multicollinearity among independent variables, which is the basic assumption of regression analysis, the Durbin-Watson statistic, tolerance, and Variance Inflation Factor (VIF) were calculated. The Durbin-Watson statistic was calculated to be 1.90–2.00, which was close to 2, confirming that there was no autocorrelation, and the tolerance was calculated to be 0.41–0.96, which was more than 0.1. The VIF was calculated to be 1.03–1.83, which was less than 10, confirming that there was no multicollinearity problem. In addition, the results of a residual analysis confirmed the linearity and homoscedasticity of the model.

The results of the three-step regression analysis are as follows. First, we observed a statistically significant negative impact of frailty on health literacy (β = −0.53, *p* < 0.001). Second, we observed that frailty had a statistically significant positive impact on medical cost (β = 0.26, *p* = 0.001). Finally, a model that considered both frailty, as an independent variable, and health literacy, as the mediator, revealed that health literacy had a statistically significant negative impact on medical cost (β = −0.04, *p* = 0.046). The model also indicated that the β value of frailty equaled 0.24, which was lower than the β value from the second analysis (0.26). Furthermore, this model highlighted the statistically significant impact of frailty on medical cost (*p* = 0.002). Therefore, our results suggest that health literacy plays a partial mediating role in the relationship between frailty and medical cost.

## 4. Discussion

Although a previous study using a systematic literature review of only nationally representative studies [25] reported that the prevalence of frailty was 7%, the rapidly growing older adult population is expected to increase the prevalence of frailty [2,18]. There is a dose-response proportional relationship between frailty and medical costs [7], and an improvement in health literacy in older adults is related to a decrease in healthcare utilization and medical costs [17]. Considering these points, efforts to manage the medical costs of older adults are urgently needed in South Korea, which has the most rapidly aging population worldwide. Therefore, this study was conducted to provide evidence for the possibility of utilizing health literacy in developing interventions to reduce medical costs for older adults by preventing and managing frailty.

The results of this study identified frailty as a factor influencing medical costs. This is consistent with the results of previous studies [7,9,26]. A previous study [27] reported that frailty among community-dwelling older adults was an important factor affecting medical costs, independent from pure age and comorbidity. South Korea is one of the countries with the most rapidly aging population worldwide [1]. In South Korea, the national health insurance expenditures for older adults aged 65 or older in 2022 accounted for 43.2% of total medical expenditures, and the average medical expenditure per older adult aged 65 or older was KRW 5.1 million, which was about 3.1 times higher than the average medical expenditures per person of KRW 1.62 million [1]. Considering the increasing prevalence of frailty due to the rapidly growing older adult population and medical costs for older adults, the active management of frailty in community-dwelling older adults is required.

The Clinical Practice Guidelines for Managing Frailty that can be used in primary care practice [26] suggest exercise and nutrition management, polypharmacy monitoring, interventions for cognitive function, fall management, and social frailty management as methods for preventing and managing frailty. In addition, a systematic literature review on interventions to prevent pre-frailty and frailty progression [28] reported that a physical exercise program conducted in a group was effective in reducing or delaying frailty. Considering the results of those previous studies, it is necessary to develop and implement programs for preventing and managing frailty in the community where older adults live, verify their effectiveness, and conduct studies to develop additional interventions.

The results of this study showed that frailty was a factor affecting health literacy. Previous studies have reported that the health literacy level decreases with age [13] and that health literacy is related to frailty progression in community-dwelling older adults [16], which is consistent with the results of this study. Although health literacy levels decrease in old age when cognitive processes decline [29], health literacy can enhance motivation and autonomy in the context of complying with frailty management strategies [30]. Therefore, improving health literacy in older adults is important. In addition, considering the increasing prevalence of frailty, the importance of health literacy in older adults should be further emphasized.

Although most previous studies have focused on the role of low health literacy in contributing to frailty, the reverse relationship—where frailty leads to diminished health literacy—has received less empirical attention. However, conditions often associated with frailty, such as cognitive decline, functional disability, and chronic illness, have been shown to negatively affect health literacy. For example, Kobayashi et al. (2015) reported that declines in cognitive function over time were significantly associated with reductions in health literacy [29]. Moreover, health literacy interventions—such as tailored education programs and active learning approaches—have shown effectiveness in populations with chronic diseases like diabetes and cardiovascular conditions. These findings suggest that frailty-related declines in function may plausibly impair health literacy and that targeted interventions could be beneficial.

The results of this study indicated that health literacy was a factor affecting medical costs. These results are consistent with those of previous studies [9,10,17]. Health literacy affects the process of understanding and developing awareness of diseases [31], and individuals with low health literacy are less likely to understand health information provided by medical professionals [12]. They may have lower health status [32], more outpatient visits and hospitalizations [15], lower self-management skills [33], and problems in using preventive medical services [32] and, thus, incur higher medical costs [15].

Low health literacy among older adults is also reported to be associated with higher mortality rates [15]. Inefficient utilization of medical services can increase related financial burdens on healthcare systems and reduce healthcare services competency, thereby lowering national competitiveness. Considering that health literacy levels can be improved through active learning programs [34], it is necessary to develop and implement such programs at the community level to improve health literacy among community-dwelling older adults. In addition, primary care institutions in the community should develop strategies to assess and respond to limited health literacy among older adult patients.

This study found that health literacy had a partial mediating effect on the relationship between frailty and medical costs. This study provided evidence for the possibility of utilizing health literacy in developing interventions to reduce medical costs for older adults by preventing and managing frailty. Health literacy is an important prerequisite for efficiently utilizing medical services [35]. Efforts to improve health literacy, together with frailty prevention and management, may help reduce medical costs in older adults, though further research is needed to confirm this relationship. In other words, education to improve health literacy should be included and strengthened in frailty intervention programs, which are focused on exercise and nutrition management, polypharmacy monitoring, interventions for cognitive function, and fall management for preventing and managing frailty. In addition, primary care providers in the community should recognize the importance of health literacy issues in older adults, provide them with information in an easy-to-understand way, and ensure that they fully understand the information. Also, recent changes in the Korean healthcare system, such as digitalization and payment reforms, may further complicate care navigation for older adults. These systemic developments highlight the increasing importance of health literacy in enabling equitable healthcare access.

This study confirmed that health literacy had a partial mediating effect on the relationship between frailty and medical costs in community-dwelling older adults using the research data that were constructed by linking the KFACD, nationally representative older adult cohort data involving community-dwelling older adults in South Korea, and the NHID. As this study used cross-sectional data, the findings reflect associations rather than causal relationships.

This study has several strengths. First, the use of linked, nationally representative data enhances the validity and generalizability of the findings. Second, the inclusion of a relatively high proportion of male participants helps address the frequent gender imbalance in aging research. Third, the study provides new insights into the mediating role of health literacy—an underexplored area in Korean aging research.

However, this study has the following limitations. First, this study included older adults aged 70–84 years, which may limit the generalizability of the findings to younger individuals aged 65–69. However, given that frailty prevalence tends to increase with age, the age range used in this study may help highlight patterns that are particularly relevant for individuals at higher risk. Second, the KFACD used in this study are survey-based data. As those who could not respond to the questionnaire due to health literacy issues might be excluded during the data collection stage, it might cause selection bias. With this in mind, the results of this study should be interpreted. Third, in the case of those who utilized healthcare services relatively little due to personal or regional medical accessibility issues, related medical costs might be estimated to be low. However, as South Korea has a national health insurance system covering all citizens, people have less burden of visiting medical institutions; the Korean government is conducting various projects to improve medical accessibility, and older adults may have relatively less difficulty using healthcare services compared to other countries [36]. However, when interpreting the results of this study, these points should be taken into account. Fourth, this study used cross-sectional data, and thus, the temporal precedence between frailty, health literacy, and medical costs could not be firmly established. It is possible that lower health literacy may precede frailty in some individuals. Therefore, the results of this study should be interpreted with caution, and longitudinal research is necessary to confirm the directional relationships and mediation effects. Fifth, although this study employed the Baron and Kenny three-step regression method due to its interpretability and its precedent in similar Korean aging cohort research, we acknowledge that this approach does not allow for direct estimation of indirect and total effects and may have limited statistical power. More advanced methods such as path analysis or bootstrap-based mediation analysis are recommended for future studies to provide more accurate and statistically robust estimates of mediation effects. Sixth, health literacy was measured using only three items derived from the Behavioral Risk Factor Surveillance System (BRFSS). Although this tool has been widely used in previous studies and has shown acceptable reliability in older adult populations, the brevity of the scale may limit its construct validity and may not fully capture the multidimensional aspects of health literacy. Future research should consider using more comprehensive and validated instruments to assess health literacy. Finally, in this study, the frailty variable was derived as an ordinal scale with three levels (robust, pre-frail, and frail) and treated as a continuous variable in the regression models. While this approach has been commonly adopted in previous studies using the KFACD to reflect a gradient of frailty severity, we acknowledge that treating a three-level ordinal variable as continuous has limitations. This simplification may not fully capture the non-linear characteristics of frailty and may reduce interpretive precision. Future research should consider applying ordinal regression models or category-specific analyses to examine the robustness of these findings.

## 5. Conclusions and Recommendations

Based on the findings of this study, it can be concluded that health literacy plays a partial mediating role in the relationship between frailty and medical costs among community-dwelling older adults. This result highlights the significance of health literacy as a meaningful factor that influences healthcare utilization. Therefore, improving health literacy in older adults may serve as an effective strategy to mitigate medical costs, particularly when combined with interventions for frailty prevention and management.

In light of these findings, it is recommended that health literacy enhancement strategies be actively integrated into frailty intervention programs at both the community and primary care levels. Primary care providers should be trained to deliver health information in ways that are accessible and easily understood by older adults. Moreover, public health efforts should promote health literacy as a modifiable and actionable factor that contributes to healthy aging and the sustainability of the healthcare system. Future longitudinal and intervention-based studies are warranted to further validate these associations and assess the effectiveness of health literacy-centered interventions.

## Figures and Tables

**Table 1:** Participants’ characteristics and frailty, health literacy, and medical cost (n = 1538).

Variable		n (%)		Frailty			Health Literacy			Medical Cost ^†^		
				M ± SD	F or t	*p*	M ± SD	F or t	*p*	M ± SD	F or t	*p*
Total		1538	(100.0)	1.89 ± 0.55			8.59 ± 2.62			1,605,633 ± 2,761,270		
Gender					7.01	0.008		143.16	<0.001		0.91	0.340
	Male	940	(61.1)	1.91 ± 0.51			9.20 ± 2.41			1,659,234 ± 2,899,373		
	Female	598	(38.9)	1.84 ± 0.60			7.63 ± 2.66			1,521,377 ± 2,529,062		
Age					37.82	<0.001		33.68	<0.001		0.26	0.769
	70–75	779	(50.7)	1.79 ± 0.52			9.04 ± 2.50			1,555,945 ± 3,013,001		
	76–80	524	(34.1)	1.91 ± 0.54			8.38 ± 2.58			1,648,467 ± 2,563,113		
	81–84	235	(15.3)	2.13 ± 0.57			7.54 ± 2.76			1,674,833 ± 2,279,902		
Spouse					00.00	0.957		101.62	<0.001		2.14	0.144
	Partnered	1123	(73.0)	1.89 ± 0.53			8.98 ± 2.50			1,543,073 ± 2,789,976		
	Single	415	(27.0)	1.88 ± 0.58			7.51 ± 2.64			1,774,922 ± 2,678,023		
Educational level					5.27	0.001		137.25	<0.001		1.67	0.173
	≤Elementary	606	(39.4)	1.95 ± 0.56			7.18 ± 2.53			1,799,380 ± 2,947,919		
	Middle school	229	(14.9)	1.85 ± 0.56			8.71 ± 2.35			1,470,268 ± 2,350,892		
	High school	338	(22.0)	1.84 ± 0.54			9.41 ± 2.24			1,455,690 ± 2,295,473		
	≥College	365	(23.7)	1.84 ± 0.51			10.07 ± 2.05			1,507,738 ± 3,050,667		
Economic activity					0.11	0.742		0.38	0.540		3.44	0.064
	In employment	384	(25.0)	1.88 ± 0.53			8.66 ± 2.65			1,379,337 ± 1,924,842		
	Unemployed	1154	(75.0)	1.89 ± 0.55			8.56 ± 2.61			1,680,934 ± 2,985,045		
Household monthly income *					8.67	<0.001		67.94	<0.001		0.15	0.931
	<100	600	(39.0)	1.97 ± 0.57			7.57 ± 2.73			1,604,381 ± 2,350,272		
	100–200	376	(24.5)	1.87 ± 0.51			8.67 ± 2.43			1,657,219 ± 2,900,015		
	200–300	245	(15.9)	1.82 ± 0.55			9.35 ± 2.24			1,636,073 ± 3,327,312		
	≥300	317	(20.6)	1.80 ± 0.51			9.81 ± 2.13			1,523,288 ± 2,841,709		
Self-rated health status					79.32	<0.001		147.66	<0.001		16.97	<0.001
	Healthy	1122	(73.0)	1.81 ± 0.52			9.06 ± 2.47			1,429,906 ± 2,598,534		
	Unhealthy	416	(27.1)	2.08 ± 0.56			7.31 ± 2.59			2,079,588 ± 3,112,941		
Smoking					1.54	0.214		28.31	<0.001		0.94	0.393
	Non-smoker	763	(49.6)	1.87 ± 0.58			8.09 ± 2.71			1,586,646 ± 2,775,808		
	Ex-smoker	668	(43.4)	1.90 ± 0.52			9.10 ± 2.40			1,677,585 ± 2,896,586		
	Smoker	107	(7.0)	1.95 ± 0.46			8.91 ± 2.66			1,291,830 ± 1,495,875		
Drinking					4.09	0.003		7.22	<0.001		6.21	<0.001
	Non-drinking	470	(30.6)	1.95 ± 0.56			8.25 ± 2.66			2,065,895 ± 3,481,819		
	≤1 time/month	450	(29.3)	1.86 ± 0.56			8.39 ± 2.58			1,574,646 ± 2,258,369		
	2–4 times/month	241	(15.7)	1.79 ± 0.53			9.17 ± 2.55			1,491,211 ± 3,404,519		
	2–3 times/week	209	(13.6)	1.88 ± 0.54			9.03 ± 2.52			1,065,710 ± 1,182,165		
	≥4 times/week	168	(10.9)	1.92 ± 0.48			8.67 ± 2.67			1,236,828 ± 1,702,256		

* Unit: 10,000 Korean won (KRW); ^†^ Unity: Korean won (KRW). M ± SD = Mean ± Standard Deviation.

**Table 2:** Correlation among frailty, health literacy, and medical cost (n = 1538).

Variable	Frailty	Health Literacy	Medical Cost
	r (*p*)	r (*p*)	r (*p*)
Frailty	1		
Health literacy	−0.27 (<0.001)	1	
Medical cost	0.15 (<0.001)	−0.07 (0.008)	1

**Table 3:** Mediating role of health literacy in the relationship between frailty and medical cost (n = 1538).

Steps	Direction	β (*p*)	t	R^2^	Adj. R^2^	F (*p*)
Step 1	Frailty → Health literacy	−0.53 (<0.001)	−4.89	0.320	0.316	79.73 (<0.001)
Step 2	Frailty → Medical cost	0.26 (0.001)		0.040	0.037	10.73 (0.001)
Step 3	Frailty, Health literacy → Medical cost			0.044	0.040	
	Health literacy → Medical cost	−0.04 (0.046)				3.96 (0.047)
	Frailty → Medical cost	0.24 (0.002)				9.34 (0.002)

Gender, age, spouse, educational level, household monthly income, self-rated health status, smoking, and drinking were adjusted in step1. Gender, age, self-rated health status, and drinking were adjusted in steps 2 and 3.

## Data Availability

The data used in this study are not open to the public. We used these data with permission from the National Evidence-Based Healthcare Collaborating Agency (NECA) and analyzed it on a computer designated by the NECA.
